# The complete chloroplast genome of *Blechnopsis orientalis* (Linnaeus) C. Presl 1753 (Blechnaceae)

**DOI:** 10.1080/23802359.2024.2385618

**Published:** 2024-08-05

**Authors:** Yu-tong Huang, Wen-xiao Men, Yan-ping Xing, Wen-juan Hou, Yan-chang Huang, Yan-yun Yang, Liang Xu

**Affiliations:** School of Pharmacy, Liaoning University of Traditional Chinese Medicine, Dalian, China

**Keywords:** Chloroplast genome, phylogeny tree, Blechnaceae, *Blechnopsis orientalis*

## Abstract

*Blechnopsis orientalis* (Linnaeus) C. Presl (1753) is a fern used both as food and medicine. It is found primarily in southern China and Southeast Asia, thriving in warm, humid shrublands or sparse forest. The total length of the chloroplast genome is 155,211 bp, including a large single-copy region (LSC, 81,877 bp), a small single-copy region (SSC, 21,500 bp), and two inverted repeat regions (IRs, 25,917 bp). The GC content is 41.3%. A total of 131 genes were annotated, including 88 protein-coding genes, eight rRNA genes, and 35 tRNA genes. The phylogenetic analysis using the maximum-likelihood method showed that *B. orientalis* and *Oceaniopteris gibba* were closely related. This study provides genomic resources for phylogenetic genetics and resource exploitation of *B. orientalis*.

## Introduction

*Blechnopsis orientalis* (Linnaeus) C. Presl (1753) is a fern species of the genus *Blechnopsis* in the Blechnaceae family. It is mainly distributed in China and parts of southeast Asia (Wang et al. 2013). In some provinces of southern China, the dry rhizomes and petiole residues of this plant are used as traditional Chinese medicine to treat diseases such as wind-heat cold, hematemesis, taeniasis, ascariasis, and so on. In some countries of Southeast Asia, it is often used to treat wounds, blisters, abscesses, and ulcers (Ahmad and Holdsworth 2003), as well as stomachache and bladder discomfort (Maridass and Ghantikumar 2008). As one of the most popular edible ferns in Asia, the main edible part is the young leaves (Piggott 1996; Huang et al. 2019). It has strong vitality and can still grow even in places with serious environmental pollution (Zhu et al. 2013). Modern pharmacological studies have found that *B. orientalis* has antioxidant, anti-cancer, and antibacterial effects. Its ethyl acetate, n-butanol, and water extracts have strong free radical scavenging activity and significant bactericidal activity against Gram-positive bacteria (Tao et al. 2009). It can be used as antioxidant with antibacterial and antitumor properties of natural materials (Lai et al. 2017).

**Figure 1.**
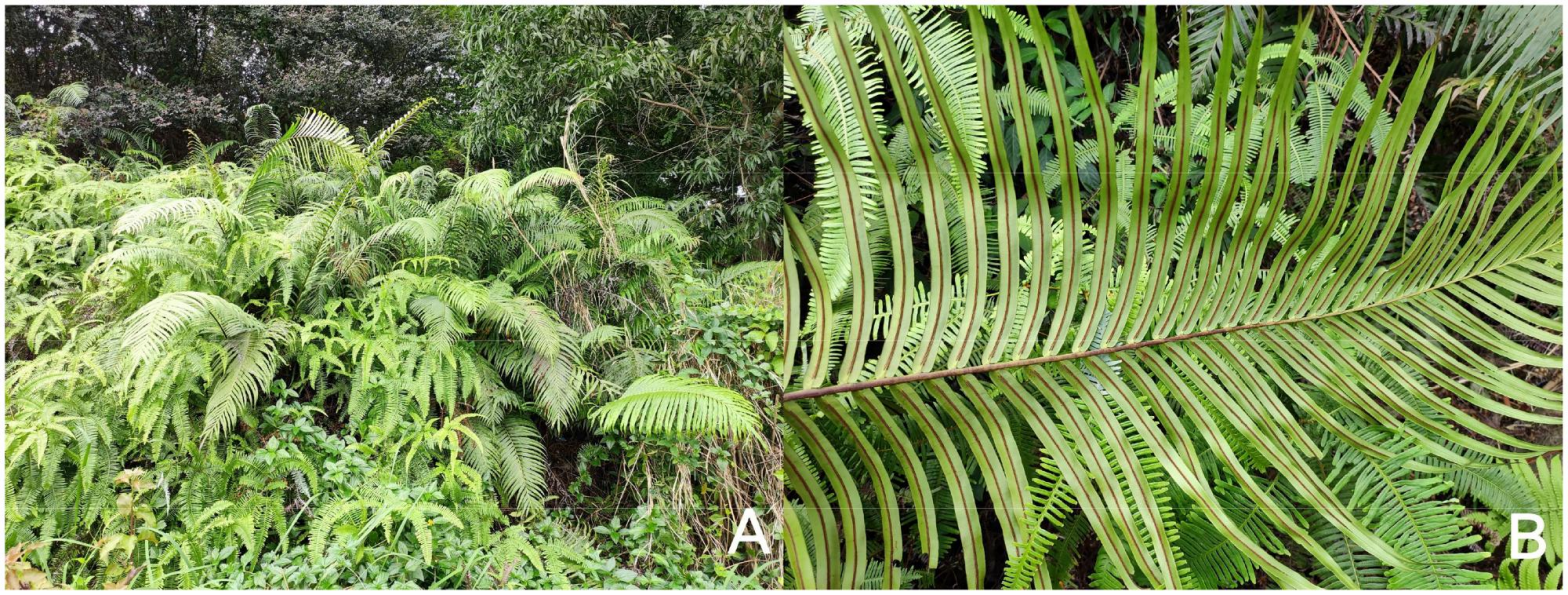
Photographs of (A) habitat and (B) linear sporangia group of *B. orientalis* taken by Ling-ling Lin in Guangzhou, Guangdong Province, China (113°21′29.40″ E, 23°9′20.11″ N). Sporangium group linear, born along both sides of the main vein, sporangium lid round, open to the main vein.

The chloroplast genome is independent of the nuclear genome and is a circular DNA consisting of four fragments: IRA, IRB, LSC, and SSC. These fragments can be used to characterize the functions and transcription processes of chloroplasts, providing information that helps influence the functionality of chloroplasts (Liu et al. 2018). Chloroplast genes have high stability and can be inherited independently, making them a reliable basis for species identification, phylogeny, origin, and evolution (Lilly et al. 2001; Liu, Ni, et al. 2023; Liu, Zeng, et al. 2023). At present, most of the studies on *B. orientalis* focus on resource distribution and pharmacological effects, lacking systematic research and analysis of its chloroplast genome and genetic background information. Therefore, in this study, the whole genome information of *B. orientalis* chloroplast was measured by Next-generation sequencing. Using bioinformatics software, we analyzed the sequence characteristics, gene composition, and phylogenetic relationship of this species. This study laid a research and theoretical foundation for the study of genetic structure and genetic diversity of *B. orientalis*, and also provided support for the phylogenetic analysis and genetic diversity research of *Blechnopsis* in the future (Figure 1).

## Materials and methods

### Plant material

Fresh leaf samples were collected from Guangzhou, Guangdong Province, China (113°21′29.40″ E, 23°9′20.11″ N) and identified by Professor Liang Xu from the Liaoning University of Traditional Chinese Medicine. A specimen was deposited at the herbarium of Liaoning University of Traditional Chinese Medicine (Liang Xu 861364054@qq.com, *B. orientalis* number: 10162231017007LY) (Supplementary Figure S1).

### DNA extraction and sequencing

Total genomic DNA was isolated from 150 mg of fresh leaves utilizing the cetyltrimethylammonium bromide technique (Doyle and Doyle 1987). Monitoring of DNA degradation and contamination was conducted using 1% agarose gels. DNA concentration was determined using the Qubit^®^ DNA Assay Kit in Qubit^®^ 3.0 Fluorometer (Invitrogen, Waltham, MA). An aliquot of the purified DNA (1 μg) underwent sonication to fragment it into pieces of 350 bp in size, which were subsequently used to build a short-insert (350 bp) library with the Nextera XT DNA library preparation kit (Illumina, San Diego, CA). The sequencing of the library was performed on the Illumina NovaSeq 6000 platform, with coverage assessment carried out through the samtools depth utility.

### Genome assembly and annotation

The raw data underwent quality filtering with the NGS QC Tool Kit v2.3.3. (https://nipgr.ac.in/ngsqctoolkit.html) (Patel and Jain 2012). And then select the high quality of the sequence data (9.47 G) and the use of assemblers SPAdes v.3.14.1 (http://cab.spbu.ru/software/spades/) from the beginning to assemble a complete chloroplast genome (Bankevich et al. 2012). Finally, the complete chloroplast genome was annotated by utilizing PGA (Qu et al. 2019) with reference to the complete chloroplast genome of *Woodwardia unigemmata* (NC028543).

### Phylogenetic analysis

To determine the position of *B. orientalis* in the phylogeny, we constructed phylogenetic trees. The complete chloroplast genomes of 23 fern species were randomly obtained from NCBI, with *Equisetum ramosissimum* as an outgroup. Common protein-coding genes of 57 chloroplast genomes were identified using MAFFT version 7.037 (Katoh and Standley 2013) and compared with *B. orientalis*. Twenty-two complete chloroplast genomes were obtained by FFT-NS-2 strategy. Finally, the phylogenetic tree of 23 chloroplast genomes was constructed based on the maximum-likelihood method with 1000 bootstrap replicates and the GTR + F + I + G4 model line selected by ModelFinder (Kalyaanamoorthy et al. 2017).

## Results

### Genome structure analysis

**Figure 2.**
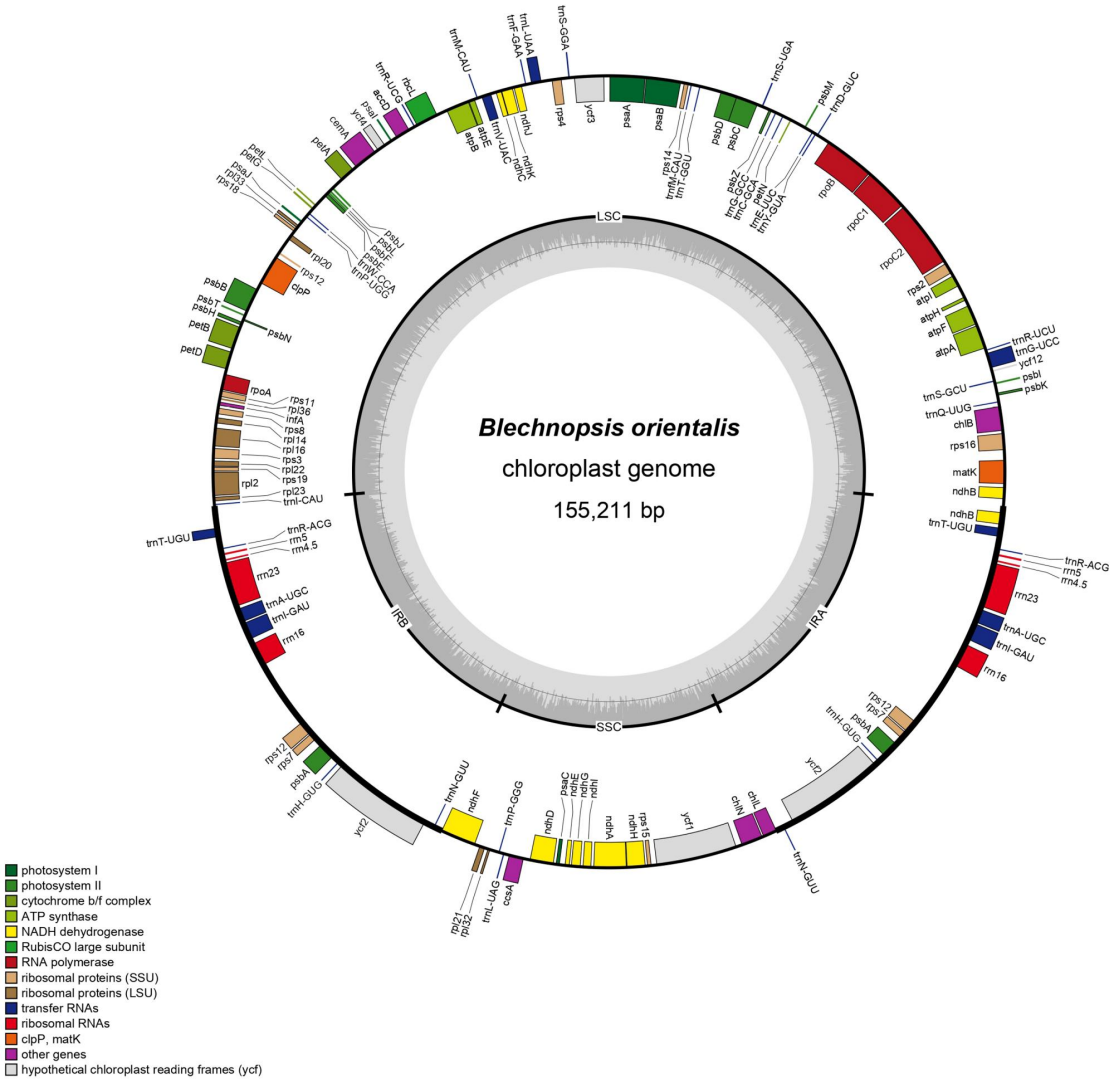
The circular map of *B. orientalis* chloroplast genome was mapped using PGA software. Genes within the ring representing transcription in a counterclockwise direction and genes outside the ring in the opposite direction. Genes with different functions were marked with different colors. The built-in gray histogram shows the genomic GC content, and the middle gray line is the 50% threshold line. Genes were colored according to their functional classification and were shown in the bottom left corner.

The total length of the chloroplast genome was 155,211 bp, including 81,877 bp of the large single-copy region, 21,500 bp of the small single-copy region, 25,917 bp of the two inverted repeat regions, and the GC content was 41.3%. A total of 131 genes were annotated, including 88 protein-coding genes, eight rRNA genes, and 35 tRNA genes. *ndh*B, *rps*16, *trn*G-UCC, *atp*F, *rpoC*1, *trn*L-UAA, *trn*V-UAC, *pet*B, *pet*D, *rpl*16, *rpl*2, *trn*I-GAU, *trn*A-UGC, *ndh*A, and *trn*T-UGU each contained one intron. The *clp*P and *ycf*3 genes contain two introns, and the *rps*12 gene is trans-spliced. The chloroplast genome of *B. orientalis* was correctly assembled according to coverage depth (Supplementary Figure S2). The maximum sequencing depth was 3532×, the minimum sequencing depth was 9×, and the average sequencing depth was 1507.77×. The annotated chloroplast genome map, cis-splicing gene map, and trans-splicing gene map (Figure 2, Supplementary Figures S3 and S4, respectively) of *B. orientalis* were processed using PAG (Qu et al. 2019) and CPGview (Liu, Ni, et al. 2023; Liu, Zeng, et al. 2023).

**Figure 3.**
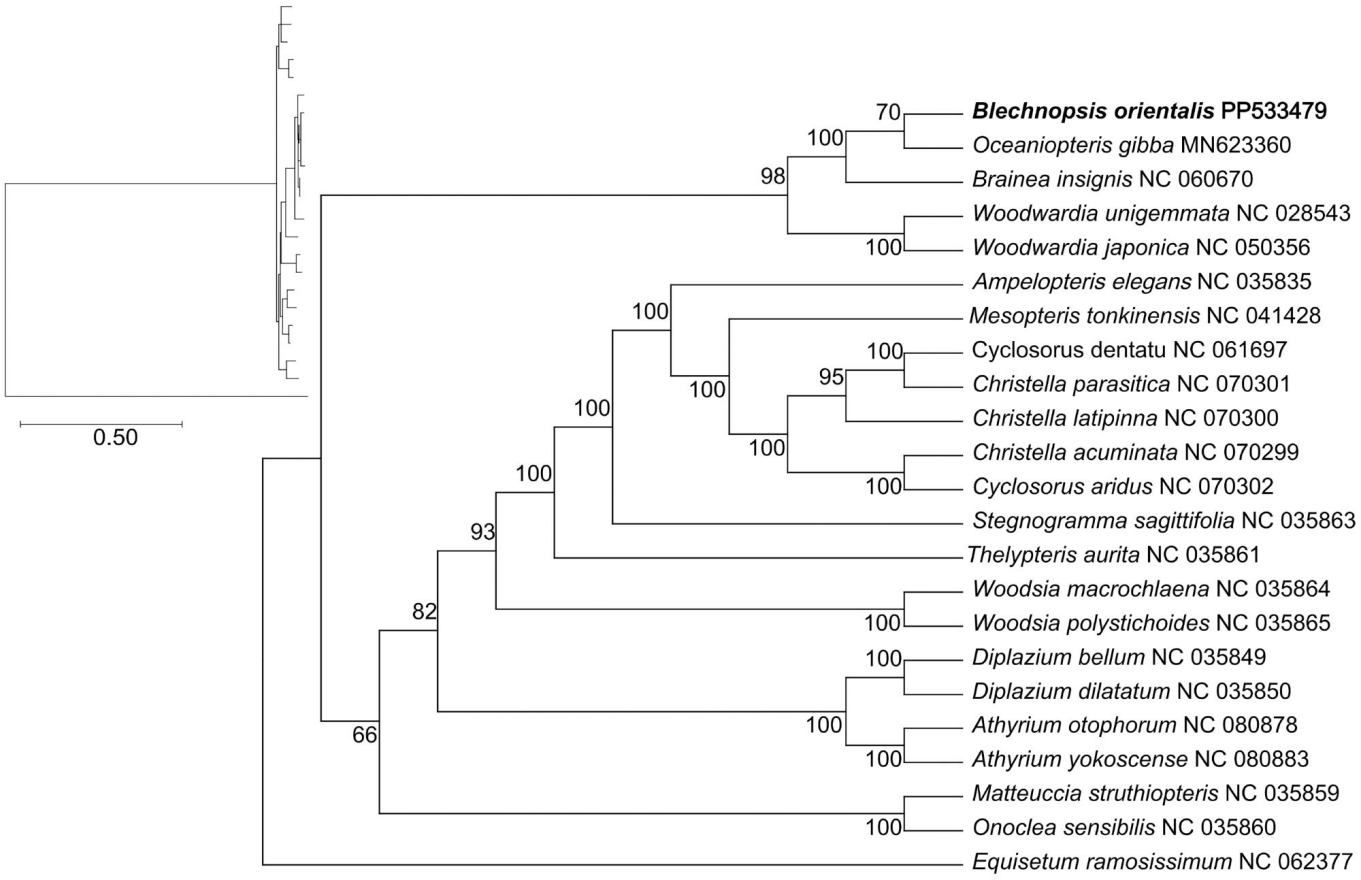
Maximum-likelihood (ML) phylogenetic tree of *B. orientalis* and 22 other complete chloroplast genome sequences. The numbers above the branches indicate the bootstrap values from ML analyses. The best evolutionary model was chosen as GTR + F + I + G4, which was selected using ModelFinder. The scale bar in the lower left corner of the figure represents the evolutionary distance, with a unit length of 0.50. The following sequences were used: *Oceaniopteris gibba* MN623360 (Liu et al. 2020), *Brainea insignis* NC060670 (Yu et al. 2020), *Woodwardia unigemmata* NC028543 (Lu et al. 2015), *Woodwardia japonica* NC050356 (Ramekar et al. 2019), *Ampelopteris elegans* NC035835 (Wei et al. 2017), *Mesopteris tonkinensis* NC041428 (Ding et al. 2018), *Cyclosorus dentatus* NC061697 (unpublished), *Christella parasitica* NC070301 (unpublished), *Christella latipinna* NC070300 (unpublished), *Christella acuminata* NC070299 (unpublished), *Cyclosorus aridus* NC070302 (unpublished), *Stegnogramma sagittifolia* NC035863 (Wei et al. 2017), *Thelypteris aurita* NC035861 (Wei et al. 2017), *Woodsia macrochlaena* NC035864 (Wei et al. 2017), *Woodsia polystichoides* NC035865 (Wei et al. 2017), *Diplazium bellum* NC035849 (Wei et al. 2017), *Diplazium dilatatum* NC035850 (Wei et al. 2017), *Athyrium otophorum* NC080878 (unpublished), *Athyrium yokoscense* NC080883 (unpublished), *Matteuccia struthiopteris* NC035859 (Wei et al. 2017), *Onoclea sensibilis* NC035860 (Wei et al. 2017), and *Equisetum ramosissimum* NC062377 (unpublished).

### Phylogenetic analysis

Phylogenetic tree analysis confirmed that *B. orientalis* and *Oceaniopteris gibba* form a monophyly branch with close genetic relationship. The establishment of phylogenetic tree will be helpful for the future research of genus *Blechnopsis* (Figure 3).

## Conclusions and discussion

In this study, we first reported the complete chloroplast genome of *B. orientalis*, which was 155,211 bp in total length and had a typical quadripartite structure. The phylogenetic tree showed that *B. orientalis* and *O. gibba* were the closest species. The Blechnaceae clustering results were consistent with previous studies (Liu et al. 2020), which provided new genetic information for analyzing the taxonomic position of *B. orientalis* in the family Blechnaceae. In addition, the complete chloroplast genome of *Blechnopsis* is rarely reported, indicating the need for further studies. The results could be used as a basis for the morphological classification of *B. orientalis*, and provide data support for the study of evolutionary relationships, species identification, and resource development of Blechnaceae. In the future, the chloroplast information of *B. orientalis* can be further applied in phylogenetic studies, identification studies, and even more drug effects can be developed through the application of the *B. orientalis* chloroplast genes in genetic engineering.

## Supplementary Material

Supplementary materials.docx

## Data Availability

The genome sequence data that support the findings of this study are openly available in GenBank of NCBI at https://www.ncbi.nlm.nih.gov/ under accession no. PP533479. The associated BioProject, SRA, and Bio-Sample numbers are PRJNA1073277, SRR27869072 (Illumina), and SAMN39824912, respectively.
