# Supplementary material for: The complete chloroplast genome of Blechnopsis orientalis (Linnaeus) C. Presl 1753 (Blechnaceae)
Source: Mitochondrial DNA B Resour. 2024 Aug 5;9(8):986–90. doi: 10.1080/23802359.2024.2385618 (PMC11302461; doi:10.1080/23802359.2024.2385618)
Supplement: Supplementary materials.docx [file TMDN_A_2385618_SM9739.docx]

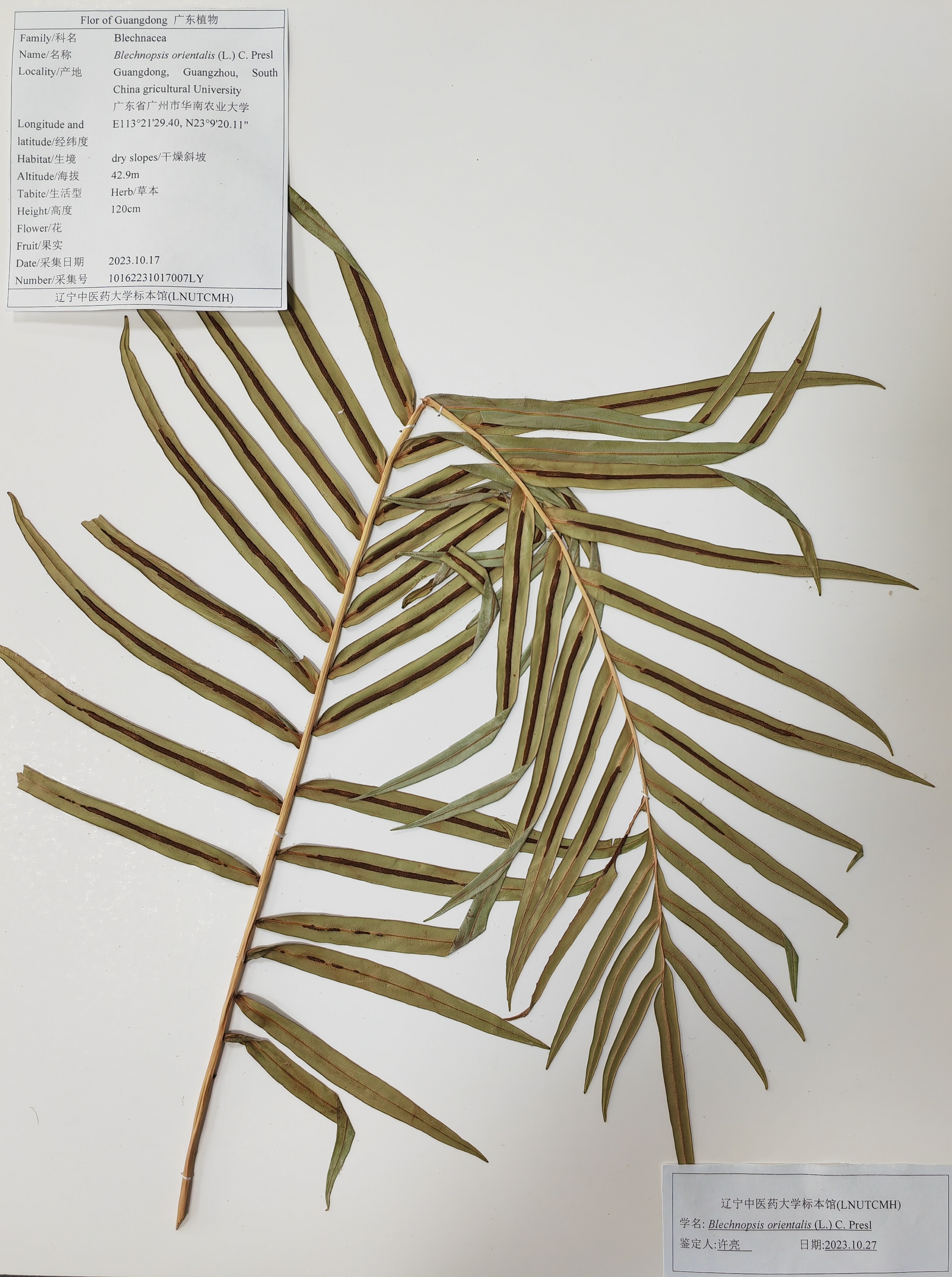


**Figure S1.** An illustration of *Blechnopsis orientalis* (L.) C. Presl specimen preserved at Liaoning University of Traditional Chinese Medicine. Specimen number, collector, latitude and longitude and other details are noted on the map for reference.


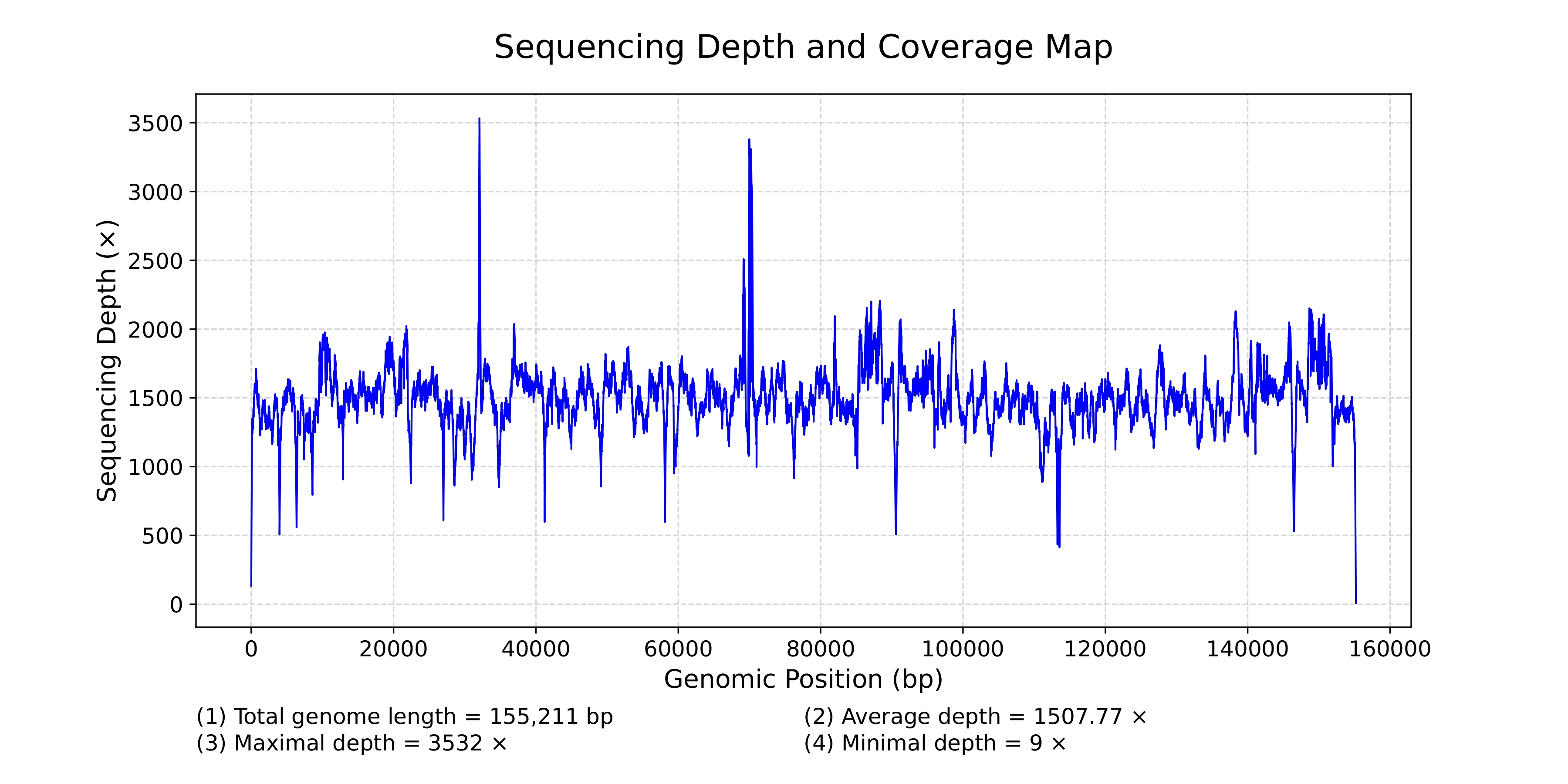


**Figure S2**. Sequencing coverage depth of *Blechnopsis orientalis* (L.) C. Presl. The illumina short sequences were compared to the chloroplast genome sequences using BWA software and finally the coverage was calculated using samtools depth (The maximum sequencing depth was 3532 X, the minimum sequencing depth was 9 X, and the average sequencing depth was 1507.77 X). The horizontal coordinate is the chloroplast length and the vertical coordinate is the coverage depth.

Li H. 2013. Aligning sequence reads, clone sequences and assembly contigs with BWA-MEM. arXiv Prepr arXiv. 0(0):3.

Li H. et al. 2009. The Sequence Alignment/Map format and SAMtools. Bioinformatics, 25, 2078-2079.


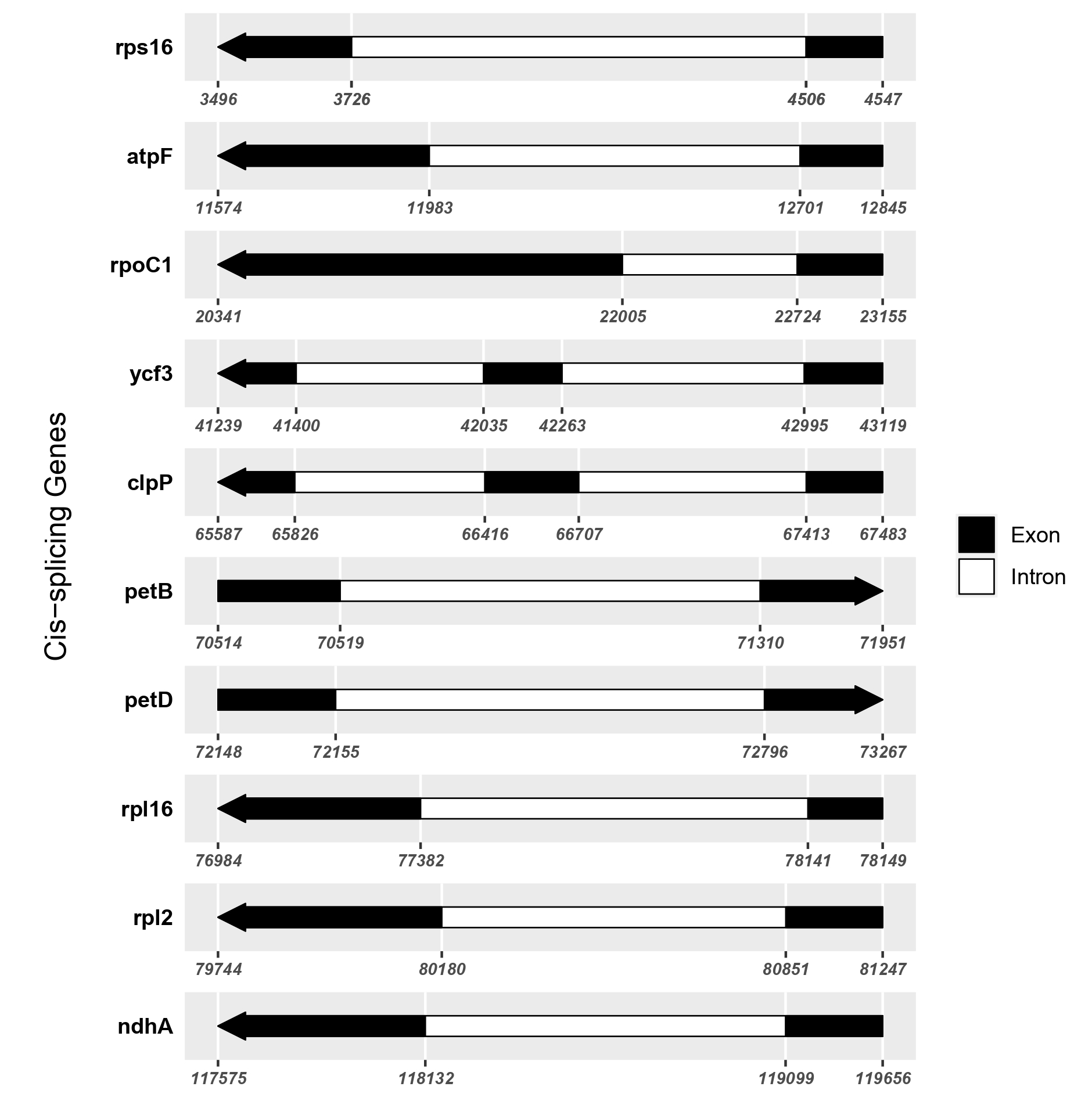


**Figure S3.** Schematic map of the cis-splicing genes in the chloroplast genome. The genes are arranged from top to bottom based on their order on the chloroplast genome. The gene names are shown on the left, and the gene structures are on the right. The exons are shown in black; the introns are shown in white. The arrow indicates the sense direction of the gene.


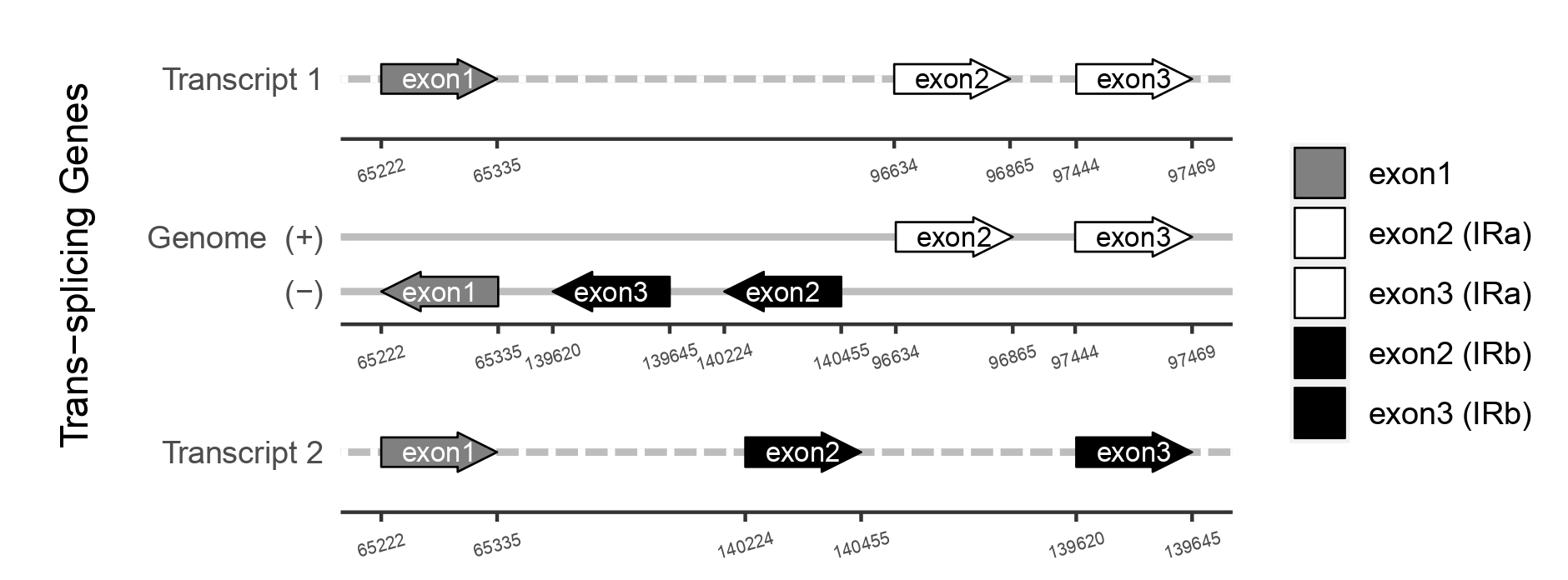


**Figure S4.** Schematic map of the trans-splicing gene *rps*12 in the chloroplast genome. It has three unique exons. Two of them are duplicated as they are located in the IR regions.
